# Improving work and employment opportunities for women with psychosocial disabilities: an action research protocol

**DOI:** 10.11604/pamj.2021.38.323.28509

**Published:** 2021-04-01

**Authors:** Isaiah Gitonga, Elena Vladimirovna Syurina, Albert Tele, Ikenna Desmond Ebuenyi

**Affiliations:** 1 Ikuze Africa, Nairobi, Kenya,; 2 Assisting Living and Learning Institute, Department of Psychology, Maynooth University, Maynooth, Ireland,; 3 Athena Institute, Faculty of Science, Vrije Universiteit, Amsterdam, The Netherlands

**Keywords:** Psychosocial disability, inclusive employment, employment support work, women, Kenya

## Abstract

In Kenya, employment rates for persons with disabilities are very low and those with psychosocial disabilities have even more dismal rates of employment. This situation has negative impact on the individual’s recovery, quality of life, mental and physical health. The systemic exclusion of persons with psychosocial disabilities in work and employment disproportionately affects women. The aim of this study is to test the feasibility of disability inclusion training to improve work and employment opportunities for women with psychosocial disabilities in Tana River County, Kenya. The study will adopt a mixed methods research design using action research approach. A sample of women with psychosocial disabilities will be trained using a researcher designed disability inclusion training manual, while employers and other stakeholders will be trained on inclusive employment. Trainings will be tailored to suit different employers and for different types of psychosocial disabilities. Interactive learning and linking sessions involving the two groups and process evaluations will be conducted at different time points to measure the impact of the intervention. Findings from this pilot study will inform future research on work and employability programs for rural women with psychosocial disabilities. The study protocol was approved by Maseno University Ethics Review Committee (MUERC/00851/20). Findings from this study will be disseminated through conference presentations and scientific publications in peer reviewed journals.

## Introduction

According to the World Health Organization (WHO) it is estimated that approximately 25% of the world´s population will experience mental illness at some point in their lives [1]. It is also well-documented that living with a mental illness can be disabling due to its impact on biological, psychological and social functioning [2]. As a result, the United Nations Convention on the Rights of Persons with Disabilities (CRPD) found it prudent to include mental impairments in its definition of disability [3]. The inclusion of mental illness in the discourses surrounding disability has been an important step in acknowledging the human rights of those with mental illnesses, specifically their rights to participate in society free from disabling social constructs. Furthermore, language surrounding mental health has started to reflect this social model of disability, for example using terms such as “person/s with a psychosocial disability” (PWPD). Social factors such as poverty, unemployment, low education, gender disadvantage and social exclusion are associated with increased burden of mental illnesses in low and middle countries. One such country is Kenya.

Recent statistics obtained from the Kenyan Ministry of Health show that approximately 1 in 4 Kenyan people have a psychosocial disability [4]; this is in keeping with the current global average [1]. However, while the mental health work force globally is on average 9 mental health professionals per 100 000 people, Kenya currently only has 0.19 mental health professionals per 100 000 people. Similarly, mental health expenditure is only 0.1% of the total health expenditure in Kenya as opposed to the global average of 2% [5]. The result is that PWPD in Kenya have limited access to resources and services that might aid in their recovery and their participation in society. Difficulties in having psychosocial disability acknowledged as a disability in Kenya are further perpetuated by a history of having outdated, neocolonialist legislation that failed to include mental health in its discussions surrounding disability [6]. Although steps have been made to advocate for PWPD through grassroots initiatives and organizations, much needs to be done in ensuring that the basic human rights of persons with psychosocial disabilities are upheld.

Unemployment is a barrier to the recovery and well-being of people living with disabilities in Kenya. As it stands only 1% of Kenyan adults with a disability are employed, compared to approximately 74% of Kenyan adults within the country´s general population, with women experiencing higher levels of systemic exclusion in work and employment than men [7, 8]. This is extremely low compared to other geographical areas [9], however it is not due to a lack of interest in work amongst PWPD. In fact, it has been reported by Boman *et al*. (2015) that PWPD are eager and motivated to work, especially for the sense of productivity, community and well-being that being employed can provide [10]. A study by Ebuenyi *et al*. made note of multiple barriers that PWPD face in gaining employment in Kenya [11]. Firstly, and most notably, the stigma surrounding psychosocial disabilities in Kenya is high. Not only does this occur at an individual and community level, but also at a national level as even national legislation concerning mental illness and disabilities uses polarizing and sometimes derogatory language. Over two thirds of a cohort of PWPD reported to have had experienced stigma and discrimination in the workplace. This has led to PWPD avoiding seeking work for fear of discrimination. Secondly, the stigma and misconceptions prevalent in society also extends to employers. It has been found that employers are often unwilling to employ PWPD for reasons such as fear for safety, concern that workplace productivity will decrease and beliefs that PWPD are unable to do basic job tasks population [8]. Consequentially few PWPD have the confidence to disclose their mental health status with their employers, meaning that they are unable to access accommodations and equity benefits that might contribute to a more conducive working environment. Thirdly, the cyclical and long-terms nature of psychosocial disabilities means that PWPD might have periods where they miss work or are able to perform their work tasks. Although this can be mitigated through proper care, the aforementioned lack of mental health professionals means that comprehensive and continual care is not an option for all PWPD. When coupled with the existing systemic unemployment burden in Kenya, it becomes clear why addressing employment for PWPD is a valuable and necessary measure.

**Base for Ikuze Africa´s proposed model:** although employment interventions for PWPD do exist, there are some gaps that need to be acknowledged and addressed. Firstly, much of the research into existing models have been conducted in high- middle income countries and therefore evidence of interventions specific to the Kenyan context is scarce. Presently, the only published research that has been done on employment for PWPD in Kenya is the work conducted by Ebuenyi *et al*. [7, 8, 11-13]. This is an important observation as it has been noted that in order for a PWPD to succeed at work, their circumstances need to be considered and support from their personal, social and political context needs to be established [14, 15]. Although studies looking at employment interventions do show promise in their results, the worker needs to be viewed as inextricably embedded in their context. This means that although interventions that work elsewhere might be equally useful in Kenya one should not assume their success is inevitable. This view is supported by the social model of disability which promotes the rhetoric that disability is a product of societal dysfunction and, since no society is exactly alike, the unique challenges faced by each PWPD needs to be addressed.

Secondly, whilst there are evidence-based strategies in place to assist in the employment of PWPD, very few interventions exist that aim to create change by engaging the employer. This is of concern as it was noted in several studies that employer support and cooperation are critical for the success of these interventions and for the sustained employment of the PWPD [16-18]. Typically, efforts to incentivize employers to employ PWPD involve legislating quota or inclusive employment systems or offering tax incentives. However, these are typically less successful in LMICs due to a lack of policing and enforcement of these laws and having a larger percentage of the population engaging in informal work. Although Kenya itself offers a 25% tax rebate to employers who employ PWPD, very few employers are aware of this [8].

The third gap that ought to be noted is that many employment interventions focus on either the PWPD or the employer, rather than on ensuring collaboration with both parties to promote mutual benefit. Many employers typically do believe that PWPD should be given equal opportunity to participate in work and do possess some knowledge of strategies on how to ensure this can be done [8]. However, employers have also been noted to lack knowledge of the nuances of mental illnesses, the support services that are available and a belief that inclusive employment is not suitable for their workplace. Interestingly, a meta-analysis by Gayed *et al*. found that by training employers in workplace mental health can improve their ability to facilitate and support employers who have psychosocial disabilities [18]. Therefore, any intervention or program that seeks to increase inclusion of PWPD in the workplace should endeavor to include employers in both their construction and their implementation to ensure success.

Lastly, it needs to be noted that although interventions that seek to increase employment for people with disabilities do exist, they often exclude PWPD. For instance, Ebuenyi *et al*. found that in one example of a technical and vocational education and training (TVET) program in East Africa, only 7% of those enrolled had a psychosocial disability [7]. This is perpetuated by legislation and societal attitudes that tend to ignore psychosocial disability as a legitimate disabling condition and exclude psychosocial disability from discourses surrounding disability and employment. We propose an inclusive, context specific intervention that will endeavor to address the highlighted gaps. The aim of the proposed study is to test the design and feasibility of a blended disability inclusive training program “the intervention” to improve employment opportunities for PWPDs in Tana River County, Kenya. We will focus on women with psychosocial disabilities as they experience even higher levels of exclusion in work and employment.

**Tana River County context:** Tana River County is one of the forty-seven counties in the Republic of Kenya. It is located approximately 700 km from Nairobi, the capital city. The County is one of the poorest in Kenya, ranked number 43 out of 47 with majority of its population living below poverty line [19]. The rate of poverty is at 76.9%, which is higher than the national average of 45.9%. The inhabitants are constantly faced with starvation, inadequate access to healthcare, low literacy levels, unemployment and insecurity.

The main economic activities are small scale farming and nomadic pastoralism [20]. There is a high rate of early girl child marriages which limits them from accessing education rights. Generally, the county has very low education enrollment [21, 22]. Boys have comparative advantage to access education over girls. Girls tend to stay away from school to do house chores, look after young children, early marriages and early pregnancies while boys look after animals and work in farms.

At the time of designing this project, Tana River County did not have a psychiatrist or psychologist. However, county is currently in the process of setting up an outpatient mental health clinic that will be run by a psychiatric nurse. There are no official statistics on number of PWPD, but the number is projected to be high considering the high levels of stigma towards mental illness and the lack of access to mental health services. This county is a representation of majority of the poor and rural counties in Kenya and therefore lessons learnt will be applicable to other similar counties.

## Methods

The proposed study will use a mixed methods design [23]. We will adopt an action research and multistakeholder approach that will ensure active participation of all stakeholders. Action research process is considered relevant and practical for finding solution to complex societal problems [24]. In addition, it ensures participation and inclusion of all stakeholders in the exploration of pathways to change. In this study, women with psychosocial disabilities, potential employers and other relevant stakeholders such as the national and county policy makers will be involved in the project design, planning and implementation. Women with psychosocial disabilities will be recruited from the general outpatient clinic at the local hospital and through the existing community-based health groups and organizations while employers from the same county will be mobilized through their local associations and umbrella employer groups.

**Needs assessment:** a needs assessment will be conducted at baseline among the women and employers to identify knowledge levels, barriers and also possible facilitators to improve employment for PWPD. Quantitative questionnaires will be administered to the women and key informant interviews will be conducted with women with psychosocial disabilities and employers from the county before the training. Needs assessment findings will be used to contextualise and adapt the training to suit the identified gaps while also borrowing on available published literature. The adapted training package will be implemented among women and the employers with special focus on inclusive employment for PWPD.

**Training:** training will be context specific and focused on the identified needs and adapted from a previous training format for youths with psychosocial disabilities in Kenya [12]. In addition to addressing these needs, training for women will cover topics related to life skills, disability awareness, rights and protections, paid and self-employment and empowerment skills, production training among others. This training will be conducted by resourceful persons with lived experience from the users and survivors of psychiatry Kenya [25] and an expert on job coaching and disability inclusion. Training package for employers and other stakeholders will include mental health, psychosocial disability, applicable disability and employment laws and policies, reasonable accommodation and workplace adjustments among others. Training for employers will be conducted by experts in job coaching and disability inclusion.

**Linking and learning:** linking and learning workshops bringing together the already trained PWPDs, employers and other stakeholders will be conducted after completion of the training. The objective of the linking and learning workshops is to provide a platform for all the stakeholders to share learnings, experiences and strategize on ways to work together towards improvement of work and employment opportunities for PWPD. During the sessions, different stakeholders will share their expectations and a common ground to balance these expectations will be sought. PWPDs will also utilize these platforms to pitch their skills and talents to the employers. Further, matching PWPD skills with employer needs, followed by a structured networking mechanism will be conducted. Ikuze Africa will conduct follow ups sessions with all the stakeholders. Immediate outcome measure will be any change in knowledge levels and attitudes in the two groups. Medium and long-term outcomes will include the number of employers expressing willingness to work and employ PWPDs, workplace and policy changes such reasonable accommodations, number of PWPD in paid employment among others. These will be measured in nine months after the intervention. Throughout the project cycle, we will conduct design and process evaluations. The project will be conducted within duration of 18 months.

**Figure 1:**
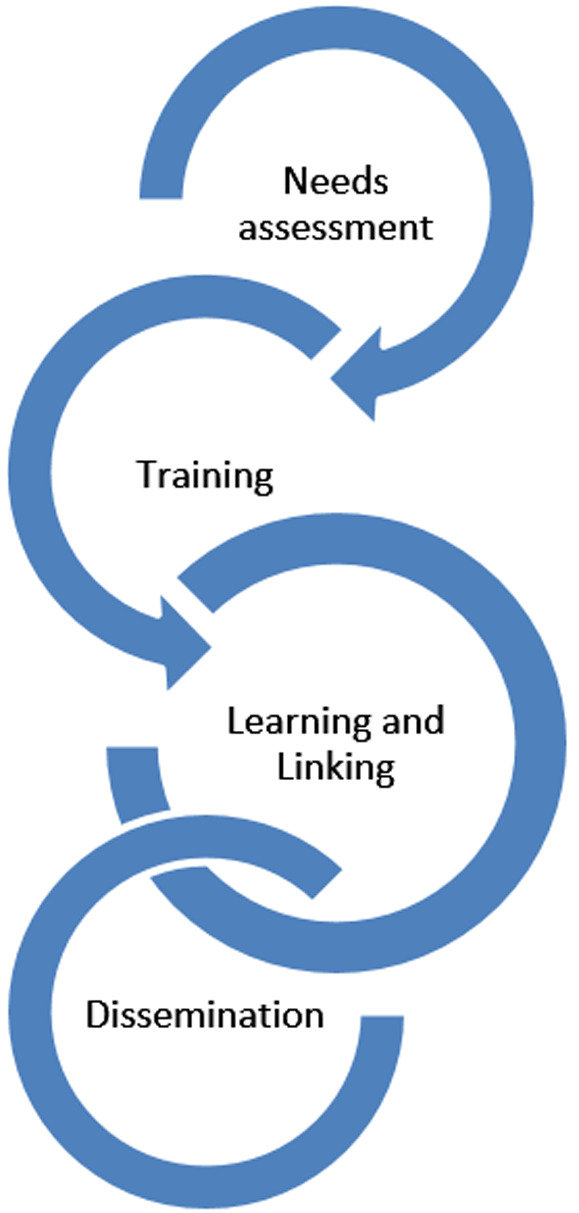
project cycle

**Dissemination of findings:** all the stakeholders would be involved in the process and outcome evaluations to ensure project ownership from inception to completion. A dissemination meeting will be organised at the end of the project and findings shared will stakeholders in Tana River County. Findings from this study will also be disseminated through scientific publications in peer reviewed journals and presentations in relevant workshops and conferences. Figure 1 summarizes the project cycle.

**Data collection:** data will be collected by means of semi structured questionnaires, interviews and focus group discussions. The Questionnaire on Anticipated Discrimination (QUAD) [26] and Mental Health Knowledge (MAKS) Schedule [27] to assess the perceptions and barriers towards seeking and initiating employment PWPD. QUAD assesses the extent to which people with mental health problems personally anticipate experiencing mental health-related discrimination across various domains of life while MAKS assesses stigma-related and disorder-specific mental health knowledge. The instruments have good psychometric properties and have been used in a variety of mental health knowledge and stigma-related studies globally, including Kenya [28-32].

Sheehan Disability Scale (SDS) [33], WHO quality of life -BREF (WHOQoL_BREF) [34] and the social functioning scale (SFS) [35] will be used to assess the changing patterns in level of disability, quality of life and social functioning respectively. The SDS is a 5-item, self-rated questionnaire that measures the extent to which a patient's disability due to illness or health problem interferes with work/school, social life/leisure activities, and family life/home responsibilities. WHOQoL_BREF is a brief measure of an individual´s perception of their position in life in the context of the culture and value systems while the SFS is a self-report questionnaire initially designed to measure the social skills and performances of patients. The tools have reported good psychometric properties and have been used widely in LMICs including Kenya [36-38]. In addition to the quantitative measurements, key informant interviews and focus group discussions (for employers, relevant stakeholders and women with psychosocial disabilities) will be conducted to assess the impact of disability inclusion training on knowledge levels, attitudes and practices of working with PWPD. They will also give an in depth understanding of the impact of the intervention on different facets such as role and social functioning and quality of life. Table 1 summarizes the study objectives, tools to measure each objective and measurement points.

**Table 1:** study objectives, data collection methods and measurement points

Specific Objective	Data Collection methods	Time points the tool(s) is administered
To understand stakeholders (employers) knowledge, attitudes and current practices in employing and working with women with psychosocial disabilities	Key informant interviews and focus group discussions with employers and stakeholders	Baseline (pre-training)
		End line (post training)
To explore perceptions and barriers towards seeking/initiating work/employment opportunities among women with psychosocial disabilities (Including their knowledge on legal protection and rights)	Questionnaire on anticipated discrimination	Baseline (pre-training)
	Mental health knowledge schedule	End line (post training)
	Key informant interviews and focus group discussions with women with psychosocial disabilities	
To test the feasibility of disability inclusion training on improving social functioning and self-esteem, stigma reduction and quality of life and disability among women with psychosocial disabilities	WHO quality of life -BREF	Baseline (pre training)
	The social functioning scales.	End line (post training)
	Sheehan´s disability scale	
	*All administered to women with psychosocial disabilities	
To test the feasibility of disability inclusion training for the purpose of improving employment opportunities of women with psychosocial disabilities	Key informant interviews and focus group discussions with employers, stakeholders, and women with psychosocial disabilities	Continuous and end line (post training)

**Data management and analysis:** all data will be stored in line with Ikuze Africa data management procedures. The first stage in the analysis of all types of variables consists of a scan of the data set to establish basic descriptive statistics that will permit a first approximation to the pattern of behavior of each variable included in the dataset. This will also help to assess the relative effectiveness and success of the data cleaning and consistency controls already executed. In the case of discrete variables, frequency tables with single or multiple cross-classification criteria will provide a good description of the variables. After the quality of the data collected has been documented and the general descriptions for the study variables have been obtained, the investigator will proceed with the statistical analysis of the whole dataset.

In the second phase of the analysis, changes between baseline and end line will be explored. Our chosen simple statistical analyses for bivariate analyses will depend on the distribution of the outcome variable and will include correlations, t- tests, chi-squared tests and their non-parametric equivalents depending on the observed distribution of the outcome variable. The results of the bivariate analyses will inform our multivariate statistical regression models for a more thorough exploration of outcome variables. Potential confounders and effect modifiers will be tested in multiple linear and logistic regression models depending on the distribution of the outcome variable. Missing data will be handled through multiple imputation techniques [23] as necessary and the level of statistical significance will be fixed at 0.05 (p<0.05) with a 95% confidence interval. Thematic analyses [23] will be used to analyze qualitative data and report the key emerging themes, which will then be corroborated with the quantitative findings.

**Ethical considerations:** the study protocol was approved by the Maseno University Ethics Review Committee (MSU/DRPI/MUERC/00851/20).

## Discussion

The aim of this proposed pilot study is to test the design and feasibility of a disability inclusion training to improve work and employment opportunities for women with psychosocial disabilities in Tana River county. It will also involve training and sensitization of employers on working with PWPD as well as training them on inclusive employment with a focus on psychosocial disabilities. Past research has emphasized the need to involve employers in interventions that target PWPD so that they can appreciate each other´s needs and balance expectations [8].

The strength of this intervention lies in collaboration of all stakeholders involved in providing employment for PWPD. First, it focuses on training and empowering women with psychosocial disabilities to waken their sense of value in the society and their inherent abilities despite their disabilities. Secondly, it sensitizes the employers through ensuring they not only have the relevant information on inclusive employment but also on benefits thereof. Thirdly, it brings together all stakeholders including the community members; the healthcare personnel and policy makers and this will ensure that the implementation is context specific and fully owned by all the stakeholders.

Involvement of all stakeholders throughout the process is informed by the fact that the existing intervention models were one sided, either focused on PWPDs only or employers only. In addition, the proposed intervention will be needs-based and context specific. It is expected that after the training, PWPD will be more engaged in both formal and informal employment. It is also expected that more employers will be willing to employ women with psychosocial disabilities. This includes their willingness and desire to make adjustments or modifications that enable PWPD to perform the essential functions of work efficiently and productively. Also important is the capacity of the intervention to motivate the women to engage is work through self-employment which studies suggest may be a realistic alternative in low income settings and globally where the informal economy [12, 39]. The success of this model is dependent on the quality of partnerships between the stakeholders (implementing organization [Ikuze Africa]; employers, PWPD, policy makers and community-based initiatives). These partnerships are critical towards the sustainability of this intervention.

## Conclusion

There are inherent gaps between available research and implementation of employment interventions for PWPDs. No such intervention has been implemented in the Kenyan context before, especially among women in the rural areas, where the burden of unemployment and stigma is highest due to little or no awareness on psychosocial disability. Successful implementation of this proposed model of supported employment and its evaluation will offer valuable lessons to reduce this gap. Furthermore, lessons learned from design to implementation process will empower Ikuze Africa, other partners and communities to plan and implement strategies which shall help to improve inclusion of PWPD in work and employment opportunities within their local contexts. In the end, increase in income through work and employment will promote successful recovery and improved quality of life for persons with psychosocial disabilities.
